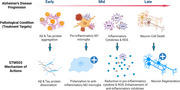# A Multi‐Target Therapy for Early and Late‐Stage Alzheimer’s Disease:Reduce Disease Progression and Restore Cognitive Functions

**DOI:** 10.1002/alz70861_108143

**Published:** 2025-12-23

**Authors:** Chun‐Ting Cheng, Bhuwnesh Agrawal, Yung‐Feng Lin, Pauline Lau

**Affiliations:** ^1^ Suntec Medical, Walnut, CA USA

## Abstract

**Background:**

Alzheimer’s disease (AD) involves multiple pathological mechanisms, including Aβ aggregation, neuroinflammation and neuronal cell death, leading to cognitive decline. We present STM‐003, a novel biologic, modulating all these mechanisms with potential to retard AD progression and improve patient cognitive function.

**Method:**

STM‐003 functions were demonstrated in 3xTg AD mice (>12 months old) after iv injection twice weekly for six weeks. Wild‐type and untreated AD mice were included as controls. Mechanism studies included Aβ and plaque reduction (Congo red staining, ELISA), microglial polarization (flow cytometry), cytokine modulation (qPCR, ELISA), and neuron viability assays in hippocampal HT‐22 cells. Brain activity and structural changes were assessed using 18F‐FDG PET and MRI. Cognitive behaviors were evaluated using the Morris water maze and tail suspension tests.

**Result:**

STM‐003 reduced plaque burden from 1.3% to 0.03%, and lowered Aβ42/40 ratio from 8.5 to 7.4 in the brain. More mechanism studies demonstrated a shift in microglia polarization from the pro‐inflammatory M1 state to the anti‐inflammatory M2 state, characterized by decreased CD14, CD86, CD80 and increased CD163, CD206 expression. Decreased TNF‐α and increased TGF‐β confirm the alleviated neuroinflammation. Additionally, STM‐003 enhanced neuronal cell proliferation by 30%, and reduced oxidative stress, and mitigated Aβ‐induced neuronal death. 18F‐FDG PET showed increased cortex and hippocampus activity (normalized uptake values: cortex from 931 to 1745, hippocampus from 838 to 1526). MRI showed 6.4% increase in the brain volume. STM‐003 improved cognitive performance shown by water maze escape latency from 56 sec to 20 sec and tail suspension inactivity from 68% to 37%.

**Conclusion:**

STM‐003 demonstrated in an AD mouse model the multifunctional efficacies in retarding the disease progression and recovering the cognitive behavior. These findings position STM‐003 as a promising therapeutic candidate for both early‐ and late‐stage Alzheimer’s disease.